# Comparative Ten-Year Outcomes in Chronic and Acute Coronary Syndrome Patients Undergoing Invasive Diagnostics—Insights from the KORONEF Registry

**DOI:** 10.3390/biomedicines12122672

**Published:** 2024-11-23

**Authors:** Adam Kern, Tomasz Stompór, Krystian Bojko, Ewa Sienkiewicz, Sebastian Pawlak, Krystyna Pawlak, Dariusz Pawlak, Grzegorz Poskrobko, Ewa Andrasz, Leszek Gromadziński, Rakesh Jalali, Dariusz Onichimowski, Grażyna Piwko, Artur Zalewski, Jacek Bil

**Affiliations:** 1 Department of Cardiology and Internal Medicine, School of Medicine, Collegium Medicum, University of Warmia and Mazury in Olsztyn, 10-727 Olsztyn, Poland; adam.kern@uwm.edu.pl (A.K.); sebastian.pawlak@uwm.edu.pl (S.P.); leszek.gromadzinski@uwm.edu.pl (L.G.); 2 Department of Cardiology, Regional Specialist Hospital in Olsztyn, 10-045 Olsztyn, Poland; stewa@mp.pl (E.S.); gposkrobko@gmail.com (G.P.); ewa8801@wp.pl (E.A.); 3 Department of Nephrology, Hypertension and Internal Medicine, School of Medicine, Collegium Medicum, University of Warmia and Mazury in Olsztyn, 10-727 Olsztyn, Poland; stompin@mp.pl; 4 Department of Monitored Pharmacotherapy, Medical University of Bialystok, 15-089 Bialystok, Poland; krystyna.pawlak@umb.edu.pl; 5 Department of Pharmacodynamics, Medical University of Bialystok, 15-089 Bialystok, Poland; dariusz.pawlak@umb.edu.pl; 6 Department of Emergency Medicine, School of Medicine, Collegium Medicum, University of Warmia and Mazury in Olsztyn, 10-727 Olsztyn, Poland; rakesh.jalali@uwm.edu.pl; 7 Clinical Emergency Department, Regional Specialist Hospital in Olsztyn, 10-045 Olsztyn, Poland; 8 Department of Anesthesiology and Intensive Care, School of Medicine, Collegium Medicum, University of Warmia and Mazury in Olsztyn, 10-727 Olsztyn, Poland; dariusz.onichimowski@uwm.edu.pl; 9 Clinical Department of Anaesthesiology and Intensive Care, Regional Specialist Hospital in Olsztyn, 10-045 Olsztyn, Poland; 10 Department of Cardiology, University of Warmia and Mazury in Olsztyn, Branch in Ełk, 19-300 Ełk, Poland; grazyna.piwko@uwm.edu.pl; 11 Scanmed Cardiology Center in Ełk, 19-300 Ełk, Poland; artur.zalewski@scanmed.pl; 12 National Medical Institute of the Ministry of Interior and Administration, 02-507 Warsaw, Poland; jacek.bil@cskmswia.gov.pl

**Keywords:** atherosclerosis, mortality predictors, invasive angiography, long-term outcomes

## Abstract

Background: This study aimed to characterize acute coronary syndrome (ACS) patients undergoing invasive diagnostics and to evaluate prognostic factors for all-cause mortality over a 10-year follow-up period. Methods: The KORONEF study was a prospective, observational, single-center study that enrolled 492 patients, of whom 467 had confirmed coronary artery disease (CAD). Baseline demographic, clinical, laboratory, and procedural data were analyzed, focusing on the differences between ACS and chronic coronary syndrome (CCS) patients. Results: Males made up the majority of both the CCS and ACS groups (62.2% vs. 63.6%, *p* = 0.773), with no statistically significant difference in patient age between the CCS and ACS subpopulations (64.9 ± 9.5 vs. 63.7 ± 10.3, *p* = 0.106). The 10-year all-cause mortality was 29.8%, with no statistically significant difference between ACS and CCS patients. However, statistically significantly more patients with CCS underwent CABG in the follow-up than ACS patients (9.9% vs. 4.6%, *p* = 0.042). In a multivariable analysis, in the ACS subgroup, statistically significant predictors of all-cause mortality at 10 years included being between 75 and 90 years old (HR 4.99), not having had a previous stroke (HR 0.27), the absence of cardiac arrest (HR 0.20), and a left ventricular ejection fraction > 60% (HR 0.23). Conclusions: The long-term outcomes of the ACS patients highlight age and left ventricular function as significant prognostic factors, underscoring the importance of these parameters in risk stratification.

## 1. Introduction

Coronary artery disease (CAD) results from restricted blood flow to the myocardium due to coronary artery blockage, primarily from plaque buildup, which disrupts the oxygen supply–demand balance. CAD remains the leading cause of mortality globally, accounting for over 9 million deaths and 185 million disability-adjusted life years, as reported by the 2021 Global Burden of Disease study, with a prevalence of approximately 1.74% [1]. While mortality from heart disease has generally decreased, recent trends show a reversal due to factors such as rising diabetes rates, aging populations, and social health disparities [2].

CAD manifests clinically as either chronic coronary syndrome (CCS) or acute coronary syndrome (ACS). ACS results from plaque rupture or erosion, leading to partial or complete arterial blockage and presenting as an ST-segment elevation myocardial infarction (STEMI), a non-ST-segment elevation myocardial infarction (NSTEMI), or an unstable angina (UA) [3]. While advancements in pharmacotherapy and interventions have improved ACS outcomes, post-hospitalization mortality remains significant. The prognosis of ACS patients varies depending on treatment, demographic factors, and comorbidities, with the highest short-term mortality rate in STEMI patients, influenced by age and sex [4,5,6].

Coronary angiography is widely used to diagnose and guide treatment decisions in CAD patients. Although essential in determining the presence and severity of stenosis, this invasive procedure carries risks, especially in patients with conditions such as acute renal insufficiency, necessitating careful benefit–risk evaluations [7].

Despite advances in managing CAD, the long-term outcomes for ACS patients vary significantly based on underlying comorbidities, treatment choices, and patient demographics. The need to accurately assess risk and optimize therapeutic strategies for ACS patients underscores the importance of comprehensive, long-term follow-up studies. Large registries, like KORONEF, might provide valuable insights into how factors such as left ventricular function, age, and coexisting conditions impact mortality and morbidity over extended periods. Such data can guide clinical decision making and highlight areas for improvement in ACS management and prevention strategies [8].

This study utilizes 10-year follow-up data from the KORONEF registry to identify mortality predictors in ACS and CCS patients, intending to enhance risk stratification and optimize patient outcomes.

## 2. Materials and Methods

### 2.1. Study Design and Participants

**Figure 1:**
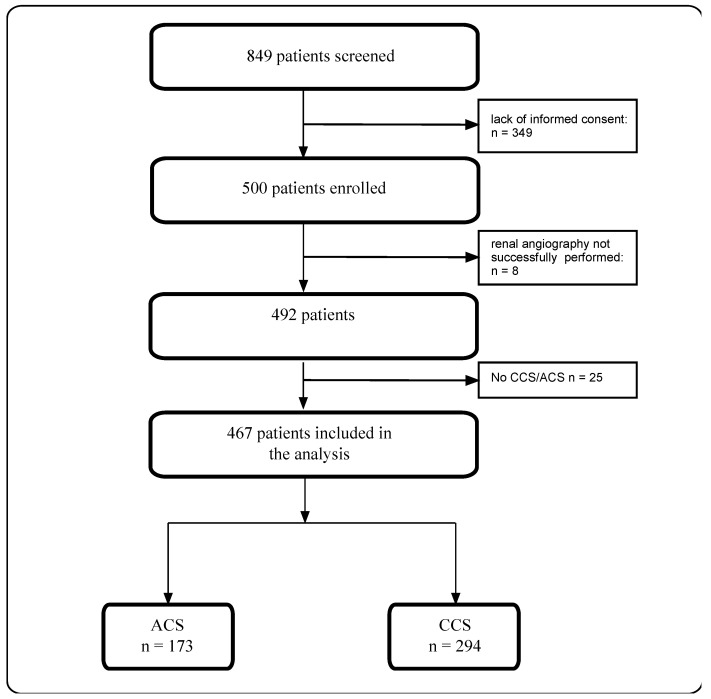
Study flowchart. ACS—acute coronary syndrome; CCS—chronic coronary syndrome.

The KORONEF study was a prospective, single-center, observational study conducted from June to December 2011. We enrolled 492 consecutive patients who underwent simultaneous coronary and renal angiography. Participants included those referred for coronary angiography due to various reasons, not only CAD diagnostics but also heart failure (HF) diagnostics, and those scheduled for other cardiovascular procedures (e.g., cardioverter defibrillator implantation, heart valve surgeries, or aortic aneurysm repair). The only exclusion criterion was the lack of informed consent. In this substudy, we only included patients with confirmed CAD (467 patients had CCS or ACS—Figure 1).

Upon admission to the cardiology department, patient histories were taken, focusing on CAD risk factors, followed by physical examinations, blood sampling, and echocardiography. All enrolled patients underwent coronary angiography along with renal artery angiography. Based on their clinical condition and CAD severity, participants qualified for conservative treatment, percutaneous coronary intervention (PCI), or coronary artery bypass grafting (CABG).

### 2.2. Data Collection

We extracted data on the following: arterial hypertension, dyslipidemia, diabetes, prior myocardial infarction (MI), history of PCI, chronic kidney disease (CKD) with an estimated glomerular filtration rate (eGFR) below 60 mL/min/1.73 m², history of CABG, peripheral artery disease, history of previous stroke, chronic obstructive pulmonary disease (COPD), and smoking status. Laboratory assessments at admission included complete blood count, creatinine, eGFR, glucose, lipid profile, thyroid-stimulating hormone, high-sensitivity C-reactive protein (hs-CRP), N-terminal pro-brain natriuretic peptide (NT-proBNP), troponin T, creatine kinase (CK), CK-MB, and uric acid. Medications prescribed at discharge were also documented.

Echocardiographic parameters were evaluated using a standard diagnostic ultrasound system. Experienced cardiologists conducted these measurements according to the European Association of Cardiovascular Imaging guidelines [9]. Follow-up data were gathered through phone call interviews. Death records were received from the Central Statistical Office (GUS) for instances of missing information.

### 2.3. Procedure Characteristics

Patients underwent coronary angiography along with simultaneous renal artery angiography. The procedure involved puncturing either the right or left femoral artery or, in select cases, the radial artery. Following the puncture, a vascular sheath was inserted to facilitate the advancement of catheters under X-ray guidance through the femoral (or radial) artery and into the aorta, reaching the ostia of coronary arteries. Diagnostic angiographic catheters with a diameter of 6F were primarily utilized for coronary angiography, mainly employing Judkins-type catheters (JL4 and JR4) and, less frequently, Amplatz-type catheters (AL1 and AR1) designed for situations in which coronary intubation is challenging. A contrast agent was injected through the catheter into the ostia of both coronary arteries. The anatomy of coronary arteries was then evaluated, focusing on the presence and location of stenosis, arterial occlusion, the characteristics of stenosis and atherosclerotic lesions, and the extent of arterial lumen narrowing.

Since coronary angiography was performed via femoral artery puncture in over 90% of cases, renal artery angiography served as a convenient addition to the examination. This step involved the administration of additional contrast medium to both renal arteries, typically around 10–20 mL, to obtain angiographic images and assess for any stenoses along their course.

### 2.4. Study Endpoints

The primary endpoint of our study was the occurrence of all-cause mortality at the 10-year mark. Secondary endpoints included the rates of MI, stroke, PCI, and CABG during the same timeframe.

### 2.5. Statistical Methods

Data characterization was conducted using descriptive statistics. The Shapiro–Wilk test evaluated the normality of continuous variables. For normally distributed data, means and standard deviations were reported, whereas medians alongside the first and third quartiles were provided for data lacking normal distribution. Categorical variables were presented as frequencies and percentages, ensuring a minimum of 95% completeness for each parameter. Group comparisons for continuous variables utilized the Student’s *t*-test for normal distributions and the Mann–Whitney test for non-normal distributions, while Fisher’s exact test assessed distributions of categorical variables. Survival analysis employed Kaplan–Meier curves, with follow-up time defined from the initial hospitalization date to either the last recorded follow-up or the date of death. Cox proportional hazards regression identified mortality predictors, with results expressed as hazard ratios (HRs) and 95% confidence intervals in univariable analysis. A multivariable Cox regression model was generated through backward stepwise selection, retaining variables with significance at the 0.1 level, and results were reported as hazard ratios (HRs) and corresponding 95% confidence intervals (CIs) [10]. Statistical analysis was performed using the R statistical package version 3.1.2 (R Core Team, 2014). For all analyses, a significance threshold of *p* < 0.05 was applied.

## 3. Results

### 3.1. Baseline Characteristics

From June to December 2011, a total of 492 patients meeting the inclusion criteria and who were free from any of the exclusion criteria were enrolled in the study. Among these participants, 467 were diagnosed with CAD, presenting clinically as chronic coronary syndrome in 294 patients and acute coronary syndrome in 173 patients. The mean age of the CAD population was 64.4 ± 9.9 years (range: 30–89 years), with a mean body mass index (BMI) of 28.0 ± 4.3 kg/m². The most prevalent comorbidities included arterial hypertension in 350 patients (74.9%), hyperlipidemia in 220 patients (47.1%), previous MI in 157 patients (31.9%), obesity in 143 patients (30.6%), nicotine dependence in 134 patients (28.7%), diabetes in 124 patients (26.6%), and a history of PCI in 105 patients (22.5%). Additionally, concomitant renal artery stenosis was identified in 59 patients (12.6%).

Males made up the majority of both the CCS and ACS groups (62.2% vs. 63.6%, *p* = 0.773), with no statistically significant difference in patient age between the CCS and ACS subpopulations (64.9 ± 9.5 vs. 63.7 ± 10.3, *p* = 0.106). The CCS group exhibited a higher prevalence of comorbidities, demonstrating statistically significant differences in the frequency of arterial hypertension (81.3% vs. 64.2%, *p* < 0.001), obesity (34.7% vs. 23.7%, *p* = 0.013), having a history of a previous MI (39.1% vs. 21.4%, *p* < 0.001), and CABG (5.8% vs. 1.7%, *p* = 0.037). In contrast, patients with ACS had a significantly higher prevalence of nicotine addiction (24.1% vs. 36.4%, *p* = 0.005) (Table 1).

The left ventricular ejection fraction in the CAD population was 52.6 ± 11.2%, and there were significant differences between the CCS and ACS subgroups (53.7 ± 11.8 vs. 51.1 ± 10.0, *p* = 0.013) (Table 1). The patients with CCS had statistically significantly lower mean values of fibrinogen (*p* = 0.012), glucose (*p* < 0.001), total cholesterol (*p* < 0.001), LDL cholesterol (*p* < 0.001), and hs-CRP (*p* < 0.001) and higher values of blood potassium (*p* < 0.001) and sodium (*p* < 0.001). Moreover, the markers of heart failure (N-terminal pro-BNP) were statistically significantly higher in the ACS subgroup (Table 2).

### 3.2. Periprocedural and Discharge Characteristics

The most common indications for coronary angiography in ACS patients were STEMI at 43.4% (*n* = 75), followed by NSTEMI at 31.2% (*n* = 54), and UA at 25.4% (*n* = 44). In 2.9% (*n* = 5) of cases, cardiac arrest was reported. Patients with ACS had one- and two-vessel disease more frequently than patients with CCS (*p* < 0.001). The most frequent lesion localizations were in the left anterior descending artery (37.3%) and the right coronary artery (34.9%). In the whole population, the number of patients referred for PCI was 211 (48.6%), which was the majority, followed by pharmacological treatment (134; 30.9%), and CABG (89; 20.5%). Additionally, patients in the ACS subgroup were significantly more likely to undergo PCI (28.9% vs. 83.4%, *p* < 0.001), while in the CCS group, pharmacological treatment was the most common therapeutic option (43.0%) (Table 3).

Patients received treatment in accordance with standards, with 94.9% of them receiving a statin at discharge, 92.9% receiving beta-blockers, 91.9% receiving acetylsalicylic acid, and 86.9% receiving an angiotensin-converting enzyme (ACE) inhibitor. In the ACS group, patients were more likely to receive acetylsalicylic acid (89.5% vs. 96.0%, *p* = 0.013), clopidogrel (51.4% vs. 90.2%, *p* < 0.001), and an ACE inhibitor (84.0% vs. 91.9%, *p* = 0.014) at discharge. Conversely, the use of an angiotensin receptor antagonist (5.8% vs. 1.7%, *p* = 0.037), calcium channel blockers (30.3% vs. 17.3%, *p* = 0.002), thiazides (12.9% vs. 5.8%, *p* = 0.014), and nitrates (21.8% vs. 11.6%, *p* = 0.006) was less frequent in this group compared to CCS patients (Table 4).

### 3.3. Ten-Year Follow-Up Data

The median follow-up period was 10.2 years (range: 5.9–10.3 years). Across the entire study population, the all-cause mortality rate was 29.8%, a myocardial infarction occurred in 12.0% of the patients, and a stroke occurred in 4.5% of the patients. No statistically significant differences were identified between the CCS and ACS subgroups for these outcomes, with the exception of CABG. Statistically significantly more patients with CCS underwent CABG in the follow-up compared to ACS patients (9.9% vs. 4.6%, *p* = 0.042) (Table 5).

**Figure 2:**
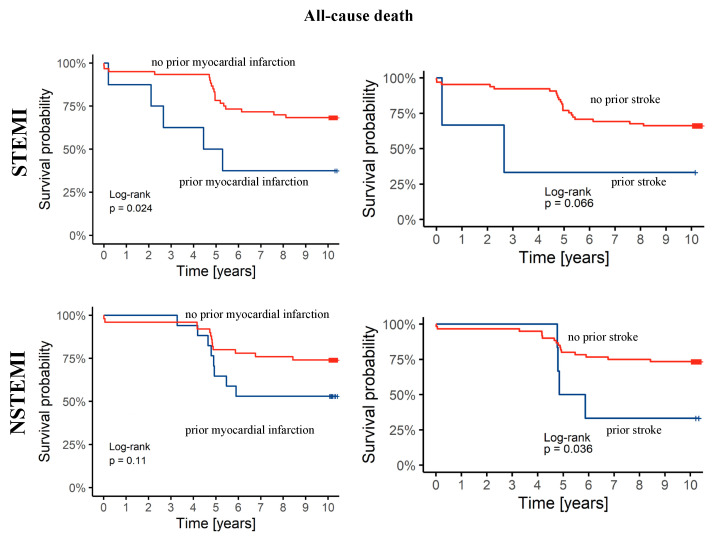

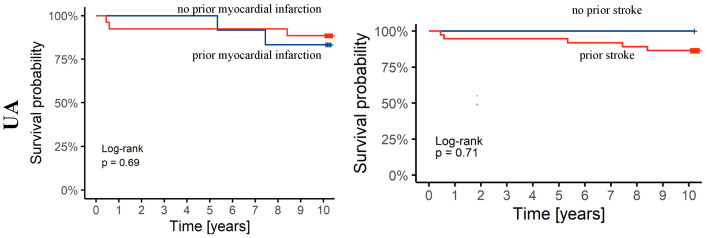
Kaplan–Meier curves showing survival depending on patients’ histories of prior myocardial infarction or prior stroke in acute coronary syndrome subtypes.

We present Kaplan–Meier curves illustrating the survival rates of additional subgroups with a history of a prior MI and a history of a prior stroke. Having a history of a prior MI had a significant impact on all-cause mortality in STEMI patients, and having a history of a prior stroke had a significant impact on all-cause mortality in NSTEMI patients (Figure 2).

### 3.4. Cox Analysis

Finally, we analyzed predictive factors for all-cause mortality at 10 years. The results of the multivariable analysis are depicted in Table 6 (univariable analyses are presented in Supplementary Tables S1 and S2).

In the subpopulation of CCS patients, statistically significant predictors of all-cause mortality at 10 years included being 75–90 years old (HR 13.3), the absence of diabetes (HR 0.41), not having had a previous MI (HR 0.46), the absence of atrial fibrillation (HR 0.04), and having had a grade-three TIMI after a PCI (HR 0.20).

For the ACS subgroup, statistically significant predictors of all-cause mortality at 10 years included being 75–90 years old (HR 4.99), not having had a previous stroke (HR 0.27), the absence of cardiac arrest (HR 0.20), and having a left ventricular ejection fraction > 60% (HR 0.23) (Table 6).

## 4. Discussion

Our study included 294 patients with CCS and 173 with ACS, with a median follow-up of 10.2 years (range: 5.9–10.3 years). In the entire cohort, the all-cause mortality rate was 29.8%, an MI occurred in 12.0% of the patients, and a stroke occurred in 4.5% of the patients. No statistically significant differences were observed between the CCS and ACS groups for these outcomes. The study population undergoing coronary angiography consisted of 37.3% women and 62.7% men, with a mean age of 64.4 ± 9.9 years (range: 30–89 years), reflecting the typical demographic profile of patients receiving invasive diagnostics for CAD. Multimorbidity was prevalent: arterial hypertension was present in 74.9%, hyperlipidemia in 47.1%, diabetes in 26.6%, a previous MI in 32.5%, obesity in 30.6%, and nicotine dependence in 28.7% of patients. These are also risk factors and/or progression factors for CAD [11,12]. Similar data can be found in the literature, including the EURECA registry [13,14,15].

An important aim of the KORONEF study was to assess the subpopulations of patients with CCS and ACS. In both groups, most of the patients were male (62.2% vs. 63.6%, *p* = 0.773). The subpopulations showed no statistically significant difference in mean age (64.9 ± 9.5 vs. 63.7 ± 10.3, *p* = 0.106) and gender distribution; however, the CCS group was characterized by a higher frequency of comorbidities, including arterial hypertension (81.3% vs. 64.2%, *p* < 0.001), obesity (34.7% vs. 23.7%, *p* = 0.013), a previous MI (39.1% vs. 21.4%, *p* < 0.001), nicotine addiction (24.1% vs. 36.4%, *p* = 0.005), and previous CABG (5.8% vs 1.7%, *p* = 0.037). Similar data can be found in the CLARIFY registry [16], which included 32,703 patients with stable CAD. The mean patient age was 64.2 ± 10.5 years, but the frequency of males was higher (77.6%). The median BMI was 27.3 kg/m^2^, indicating that most of the subjects were overweight or obese. Most of the patients had dyslipidemia (74.9%) arterial hypertension (71.0%), and were either current (12.5%) or former (46.2%) smokers. Kite et al. [17] analyzed ACS patients with COVID-19 who underwent invasive coronary angiography. Of the 316 patients, 144 (54.3%) were diagnosed with STEMI and 121 (45.6%) with NSTE-ACS. The mean age of the STEMI/NSTE-ACS combined cohort was 64.9 ± 12.9 years, 75.5% were men, 66.2% had hypertension, 54.1% had hyperlipidemia, 36.2% had diabetes mellitus, 20.2% had a previous MI, 19.3% had a prior history of heart failure, 14.6% had chronic kidney disease stage three to five, and 27.1% were current smokers. Also, another study based on the ACSIS registry [18], evaluating 5359 ACS patients, described baseline characteristics quite similar to our study. The study included patients with Thrombolysis in Myocardial Infarction Risk Score for Secondary Prevention (TRS2°P) data. Depending on the risk stage, the mean age was 69, 75, and 77 years old (corresponding to the high-risk, very high-risk, and extremely high-risk groups, respectively). Most of the patients were males (69.7%, 69.1%, and 71.8%), and the most frequent comorbidities were hypertension (87.7, 92.4%, and 97.3%), diabetes (57.1%, 69.4, and 80.7%), dyslipidemia (74.1%, 77.5%, and 82.3%), and a prior MI (38.1%, 51.9%, and 65.5%). The older age and higher frequency of comorbidities compared to our study may be explained by the selective inclusion criteria (for patients with high, very high, and extremely high levels of risk for recurrent cardiovascular events); however, it highlights the most common risk factors.

The KORONEF study enrolled patients in 2011, which likely accounts for the relatively high rates of CABG (19.7%) and PCI (46.1%). Additionally, clopidogrel was the sole P2Y12 receptor inhibitor used, as ticagrelor and prasugrel, which are now recommended for ACS patients, were not yet standard practice [19]. This could have had an impact on the prognosis. The LAD was the most common site of coronary lesions in the CAD population (36.1%), with statistically significant differences between the CCS and ACS groups (27.7% vs. 41.7%). Similar findings were reported by Hamza et al. [20], in which the LAD was a localization of the culprit lesion in 46–48% of ACS patients. The CCS and ACS subpopulations showed statistically significant differences in single-vessel and multivessel disease prevalence. The ACS subpopulation had a higher frequency of one-vessel (43.4%) and two-vessel disease (39.6%) than CCS patients. However, multivessel disease, including three vessels, was more frequent in the CCS group (22.7%). According to Toma et al. [21], patients with multivessel disease had a higher-baseline-risk profile that included the following characteristics: older age, male sex, and a higher prevalence of diabetes, hypertension, prior MI, chronic kidney disease, and left ventricular systolic dysfunction. Regarding the clinical presentation of CAD patients, the KORONEF study exhibited STEMI as the most common indication for coronary angiography in the ACS group. These outcomes are consistent with data obtained from a combined retrospective/prospective study published by Maroszyńska-Dmoch et al. [22], which evaluated clinical and angiographic characteristics of young adults with CAD and assessed mortality in a study population of 239 patients aged 40 years or younger. The outcomes showed that the most common presentation of acute coronary syndrome (ACS) in this population was STEMI (52.8%). The most common location of significant atherosclerotic coronary lesions was the LAD (61.6%), followed by the right coronary artery (27.4%). However, it should be noted that this study was conducted on a young patient population.

The baseline population and prognosis may also differ depending on the type of ACS, as demonstrated in several studies. Fan Ye et al. [4] showed that patients with STEMI face a higher risk of all-cause mortality during a short-term follow-up period (30 days). At the same time, those with NSTEMI exhibited a higher mortality rate compared to individuals with stable CAD at a two-year follow-up. These results may be linked to age-related variations. The STEMI patients were generally younger, with fewer comorbidities and fewer prior cardiac symptoms before the onset of the STEMI.

In contrast, the NSTEMI and stable CAD patients were older and had more advanced coronary lesions. Early clinical presentations in younger patients often lead to a prompt diagnosis, appropriate pharmacotherapy, and a thorough follow-up. Another interesting finding was the significant role of sex differences in the ACS patient population. Female STEMI patients had significantly higher rates of all-cause and cardiac death than their male counterparts during a two-year follow-up period. Conversely, there were no sex differences in all-cause or cardiac death rates among patients with NSTEMI and stable CAD. Another study by Toyoda et al. [23] presented similar conclusions regarding the long-term prognosis for ACS subtypes. This comprehensive Japanese multicenter registry, encompassing 3283 patients with acute MI, specifically examined three subgroups: STEMI, NSTEMI with elevated creatine kinase (NSTEMI+CK), and NSTEMI without creatine kinase elevation (NSTEMI-CK). Prognostic factors differed significantly across these subgroups, with both the NSTEMI+CK and NSTEMI-CK groups exhibiting poorer long-term (three-year) outcomes compared to STEMI patients. Regarding UA as another clinical presentation of ACS, Piątek et al. [24] analyzed a registry of 7187 patients who underwent PCI for an STEMI, NSTEMI, UA, or stable angina. The analysis revealed substantial differences among the groups in mortality rates and the risk of cardiovascular events. Over a three-year observation period, the risk of death, MI, and MACE in the UA group post-PCI was higher than in the stable angina group but considerably lower than in the NSTEMI or STEMI groups. A multivariable analysis confirmed that the prognosis in the UA group was significantly better than in the NSTEMI and STEMI groups. These results are consistent with other recent studies [25,26,27]. The influence of different age-based populations on the prognosis of patients with ACS is supported by the results of the KORONEF analysis. Our study identified risk factors for an unfavorable prognosis over the 10-year follow-up period. This analysis showed a significant inverse correlation between being of an older age and long-term mortality in the general study population, as well as in both the ACS and CCS subpopulations.

However, no statistically significant differences were found between the ACS and CCS subpopulations in terms of death, MI, and stroke occurrence over a 10-year follow-up period. In the patients with CCS, the multivariable Cox regression model identified significant predictors of all-cause mortality, including the absence of atrial fibrillation (HR 0.04), having a grade-three TIMI post-PCI (HR 0.20), not having had a prior MI (HR 0.46), the absence of diabetes (HR 0.41), and being 75–90 years old (HR 13.3). For the patients with ACS, significant predictors included a left ventricular ejection fraction > 60% (HR 0.23), the absence of cardiac arrest (HR 0.20), not having had a stroke (HR 0.27), and being 75–90 years old (HR 4.99). The median follow-up period was 10.2 years (range: 5.9–10.3 years). In the CAD population, the all-cause mortality was 29.8%, an MI occurred in 12.0% of the patients, and a stroke occurred in 4.5% of the patients. Similar outcomes were observed in patients with MINOCA and STEMI in a study with a 9-year follow-up period (17.9% vs. 24.1%, HR 1.15, 95% CI 0.67–1.94, *p* = 0.61) [28]. Muller et al. reported slightly higher values in their 5-year study, in which the decision between PCI and CABG for proximal LAD stenosis was based on the FFR result. In the conservatively treated group, the percentage of deaths was 5.3%, the occurrence of an MI was 0.4%, and the frequency of repeated revascularization was 2.0%. Conversely, in the invasively treated group, the death rate was 9.6%, the occurrence of an MI was 1.2%, and the frequency of repeated revascularization was 15.9% [29]. Similar results over 7 years were reported by Yamashita et al., with the percentage of cardiovascular events ranging from 27% to 33% [30]. Goy et al. documented 10-year outcomes of the randomized SIMA study comparing PCI with BMS versus CABG using the left internal mammary artery (LIMA) for proximal LAD stenosis, showing lower death rates and MI percentages than in our population (the death rates were 8.1% vs. 6.8%, *p* = 0.4, and the MI rates were 4.8% vs. 5.1%, *p* = 0.9), although their study had a significantly smaller sample size (62 and 59 patients) [31]. Table 7 presents the summary of outcomes in ACS patients.

### Study Limitations

This study had several limitations. The inclusion criteria encompassed patients with CAD and all individuals undergoing coronary angiography, which contributed to a lack of homogeneity in the study population. However, this broader approach allowed for an evaluation of RAS prevalence beyond those with advanced CAD. Also, the use of clopidogrel exclusively, rather than more recent P2Y12 receptor inhibitors, may have negatively influenced patient prognosis.

## 5. Conclusions

The population of CAD patients referred for coronary angiography was predominantly male, aged over 60, with multiple comorbidities, including arterial hypertension, hyperlipidemia, and a previous MI. This CAD population exhibited a significant risk of major adverse events, such as death, MI, and stroke, with similar long-term event rates for CCS and ACS groups. The ACS group had a higher prevalence of nicotine addiction and lower left ventricular ejection fraction compared to the CCS group. Most ACS patients presented with an STEMI treated with PCI and often had one- or two-vessel disease, primarily in the left anterior descending artery and right coronary artery. Over a 10-year follow-up period, the all-cause mortality rate was 29.8%, with significant predictors of mortality including being of an older age and having a left ventricular ejection fraction greater than 60%.

## Figures and Tables

**Table 1:** Baseline characteristics.

Parameter	Total Study Population *N* = 467 (%)	Patients with CCS *N* = 294 (%)	Patients with ACS *N* = 173 (%)	*p*
Females	174 (37.3%)	111 (37.8%)	63 (36.4%)	0.773
Age (years)	64.4 ± 9.9	64.9 ± 9.5	63.7 ± 10.3	0.106
BMI (kg/m^2^)	28.0 ± 4.3	28.3 ± 4.4	27.4 ± 4.0	0.077
Arterial hypertension	350 (74.9%)	239 (81.3%)	111 (64.2%)	<0.001
Hyperlipidemia	220 (47.1%)	147 (50.0%)	73 (42.2%)	0.103
Diabetes	124 (26.6%)	85 (28.9%)	39 (22.5%)	0.132
Obesity	143 (30.6%)	102 (34.7%)	41 (23.7%)	0.013
Nicotine addiction	134 (28.7%)	71 (24.1%)	63 (36.4%)	0.005
Positive family history	72 (15.4%)	46 (15.6%)	26 (15.0%)	0.858
Previous MI	152 (32.5%)	115 (39.1%)	37 (21.4%)	<0.001
Previous stroke	31 (6.7%)	21 (7.1%)	10 (5.8%)	0.579
Peripheral artery disease	25 (5.1%)	23 (5.0%)	2 (5.7%)	0.696
Dialysis	4 (0.9%)	4 (1.4%)	0 (0.0%)	0.302
Chronic kidney disease	45 (9.6%)	31 (10.5%)	14 (8.1%)	0.386
Aortic aneurysm	3 (0.6%)	2 (0.7%)	1 (0.6%)	>0.999
Renal artery stenosis	59 (12.6%)	36 (12.2%)	23 (13.3%)	0.742
Previous heart valve surgery	3 (0.6%)	2 (0.7%)	1 (0.6%)	>0.999
Previous CABG	20 (4.3%)	17 (5.8%)	3 (1.7%)	0.037
Previous PCI	105 (22.5%)	69 (23.5%)	36 (20.8%)	0.506
Echocardiography Results				
EF (%)	52.6 ± 11.2	53.7 ± 11.8	51.1 ± 10.0	0.013
TAPSE (mm)	17.4 [12.0–23.0]	20.5 [18.0–23.0]	16.2 [12.0–22.0]	0.329

Results presented as mean ± standard deviation or median (interquartile range); BMI—body mass index; MI—myocardial infarction; CABG—coronary artery bypass grafting; PCI—percutaneous coronary intervention; EF—ejection fraction; TAPSE—tricuspid annular plane systolic excursion.

**Table 2:** Biochemical tests.

Parameter	Total Study Population *N* = 467 (%)	Patients with CCS *N* = 294 (%)	Patients with ACS *N* = 173 (%)	*p*
Erythrocytes (10^12^/L)	4.7 [4.3, 5.0]	4.7 [4.3, 5.0]	4.6 [4.4, 5.0]	0.753
Hemoglobin (g/dL)	13.9 [5.9–18.2]	13.9 [5.9–18.2]	13.8 [8.6–16.6]	0.997
Hematocrit (%)	40.8 [14.6–52.7]	41.0 [14.6–52.7]	40.5 [27.8–48.9]	0.298
Fibrinogen (mg/dL)	398.5 [104.0–834.0]	389.4 [104.0–834.0]	419.6 [198.0–809.0]	0.012
Glucose (mg/dL)	117.3 [74.0–406.0]	111.2 [74.0–275.0]	127.7 [75.0–406.0]	<0.001
Creatinine (mg/dL)	1.0 [0.3–9.1]	1.0 [0.5–9.1]	0.9 [0.3–2.5]	0.322
Blood urea nitrogen (mg/dL)	38.4 [13.0–161.0]	38.5 [16.0–138.0]	38.2 [13.0–161.0]	0.356
K^+^ (mmol/L)	4.4 [2.8–6.5]	4.5 [3.1–6.0]	4.2 [2.8–6.5]	<0.001
Na^+^ (mmol/L)	140.7 [131.0–152.0]	141.3 [131.0–152.0]	139.6 [131.0–146.0]	<0.001
Cl^−^ (mmol/L)	103.4 [5.9–196.1]	103.9 [92.2–196.1]	102.5 [5.9–114.0]	0.087
Total cholesterol (mg/dL)	183.7 [70.0–607.0]	175.4 [70.0–320.0]	198.3 [79.0–607.0]	<0.001
LDL (mg/dL)	110.1 [28.0–465.0]	103.2 [28.0–241.0]	122.1 [34.0–465.0]	<0.001
HDL (mg/dL)	53.3 [17.0–183.0]	52.8 [17.0–132.0]	54.0 [22.0–183.0]	0.589
Triglycerides (mg/dL)	118.0 [86.0,163.8]	119.0 [87.0,161.0]	116.0 [86.0,169.0]	0.945
TSH (μIU/mL)	2.0 [0.0–58.6]	1.8 [0.0–11.1]	2.6 [0.0–58.6]	0.052
NT-proBNP1 (pg/mL)	1622.5 [0.0–70,000]	1480.0 [0.0–70,000]	1762.0 [0.5–33,029]	0.003
hs-CRP (mg/L)	1.2 [0.0–82.0]	0.4 [0.0–10.2]	2.5 [0.0–82.0]	<0.001

Results presented as median (interquartile range); HDL—high-density lipoprotein; LDL—low-density lipoprotein; TSH—thyroid-stimulating hormone; NT-proBNP—N-terminal prohormone of brain natriuretic peptide; hsCRP—high-sensitivity C-reactive protein.

**Table 3:** Periprocedural data.

Parameter	Whole Study Population *N* = 467 (%)	Patients with CCS *N* = 294 (%)	Patients with ACS *N* = 173 (%)	*p*
Coronary angiography results				
One-vessel disease	135 (37.3%)	66 (32.5%)	69 (43.4%)	<0.001
Two-vessel disease	136 (37.6%)	73 (36.0%)	63 (39.6%)	
Three-vessel disease	66 (18.2%)	46 (22.7%)	20 (12.6%)	
Left main stem	22 (6.1%)	18 (8.9%)	4 (2.5%)	
Qualification for revascularization				
Pharmacological treatment	134 (30.9%)	119 (43.0%)	15 (9.6%)	<0.001
PCI	211 (48.6%)	80 (28.9%)	131 (83.4%)	
CABG	89 (20.5%)	78 (28.2%)	11 (7.0%)	
Location of lesions treated by PCI				
Bypass	2 (0.8%)	2 (2.1%)	0 (0.0%)	0.017
Left circumflex artery/marginal branch	59 (24.7%)	28 (29.8%)	31 (21.5%)	
Intermediate artery	4 (1.7%)	4 (4.3%)	0 (0.0%)	
Left anterior descending artery/diagonal branch	89 (37.3%)	28 (29.9%)	61 (42.4%)	
Left main stem	1 (0.4%)	0 (0.0%)	1 (0.7%)	
Right coronary artery	83 (34.9%)	32 (34.0%)	51 (35.4%)	
Number of implanted bare metal stents				
0	321 (68.7%)	240 (81.6%)	81 (46.8%)	<0.001
1	121 (25.9%)	48 (16.3%)	73 (42.2%)	
2	21 (4.5%)	6 (2.0%)	15 (8.7%)	
3	4 (0.9%)	0 (0.0%)	4 (2.3%)	
Number of implanted drug-eluting stents				
0	380 (81.5%)	258 (87.8%)	122 (70.9%)	<0.001
1	77 (16.5%)	31 (10.5%)	46 (26.7%)	
2	8 (1.7%)	4 (1.4%)	4 (2.3%)	
3	1 (0.2%)	1 (0.3%)	0 (0.0%)	
TIMI after PCI				
0	11 (4.6%)	3 (3.2%)	8 (5.5%)	0.876
1	3 (1.3%)	1 (1.1%)	2 (1.4%)	
2	1 (0.4%)	0 (0.0%)	1 (0.7%)	
3	225 (93.8%)	90 (95.7%)	135 (92.5%)	
Periprocedural complications (PCI)				
No reflow/slow reflow	9 (2.1%)	3 (1.1%)	6 (3.6%)	0.120
Stent thrombosis	1 (0.2%)	1 (0.4%)	0 (0.0%)	

TIMI—thrombolysis in myocardial infarction; PCI—percutaneous coronary intervention; CABG—coronary artery bypass grafting.

**Table 4:** Medications at discharge.

Parameter	Total Study Population *N* = 467 (%)	Patients with CCS *N* = 294 (%)	Patients with ACS *N* = 173 (%)	*p*
Acetylsalicylic acid	429 (91.9%)	263 (89.5%)	166 (96.0%)	0.013
Clopidogrel	307 (65.7%)	151 (51.4%)	156 (90.2%)	<0.001
ACE inhibitor	406 (86.9%)	247 (84.0%)	159 (91.9%)	0.014
Angiotensin receptor antagonist	20 (4.3%)	17 (5.8%)	3 (1.7%)	0.037
Beta-blocker	434 (92.9%)	268 (91.2%)	166 (96.0%)	0.051
Ca-blocker	119 (25.5%)	89 (30.3%)	30 (17.3%)	0.002
Statins	443 (94.9%)	277 (94.2%)	166 (96.0%)	0.412
Fibrates	15 (3.2%)	10 (3.4%)	5 (2.9%)	0.762
Loop diuretic	80 (17.1%)	51 (17.3%)	29 (16.8%)	0.872
Thiazide	48 (10.3%)	38 (12.9%)	10 (5.8%)	0.014
Mineralcorticoid receptor antagonist	51 (10.9%)	30 (10.2%)	21 (12.1%)	0.517
Alpha-blocker	12 (2.6%)	9 (3.1%)	3 (1.7%)	0.548
Oral anticoagulation	33 (7.1%)	23 (7.8%)	10 (5.8%)	0.405
Insulin	44 (9.4%)	28 (9.5%)	16 (9.2%)	0.922
Nitrates	84 (18.0%)	64 (21.8%)	20 (11.6%)	0.006

ACE—angiotensin-converting enzyme.

**Table 5:** Ten-Year follow-up outcomes.

Endpoint	CAD Study Population *N* = 467 (%)	Patients with CCS *N* = 294 (%)	Patients with ACS *N* = 173 (%)	*p*
Death	139 (29.8%)	89 (30.4%)	50 (28.9%)	0.737
MI	56 (12.0%)	29 (9.9%)	27 (15.6%)	0.067
Stroke	21 (4.5%)	12 (4.1%)	9 (5.2%)	0.578
CABG	37 (7.9%)	29 (9.9%)	8 (4.6%)	0.042
PCI	79 (17.0%)	50 (17.1%)	29 (16.8%)	0.933

MI—myocardial infarction; CABG—coronary artery bypass grafting; PCI—percutaneous coronary intervention.

**Table 6:** Factors predicting death at the 10-year follow-up—multivariable Cox analysis.

	Study Population			Patients with CCS			Patients with ACS		
Parameter	HR	95% CI	*p*-Value	HR	95% CI	*p*-Value	HR	95% CI	*p*-Value
Age									
[30, 55]	—	—	—	—	—	—	—	—	—
[55, 60]	2.12	0.92, 4.86	0.076	5.29	0.63, 44.2	0.12	0.94	0.30, 2.97	>0.9
[60, 65]	1.24	0.48, 3.16	0.7	3.04	0.33, 27.7	0.3	0.54	0.13, 2.34	0.4
[65, 75]	2.88	1.31, 6.34	0.008	3.97	0.48, 32.6	0.2	1.60	0.54, 4.69	0.4
[75, 90]	8.07	3.65, 17.8	<0.001	13.3	1.68, 106	0.014	4.99	1.70, 14.7	0.003
Diabetes									
no	0.63	0.42, 0.95	0.028	0.41	0.19, 0.87	0.021	—	—	—
Previous myocardial infarction									
no	0.61	0.41, 0.92	0.017	0.46	0.22, 0.98	0.045	—	—	—
Previous stroke									
no	—	—	—	—	—	—	0.27	0.11, 0.68	0.005
Cardiac arrest									
no	—	—	—	—	—	—	0.20	0.04, 0.95	0.044
Atrial fibrillation									
no	0.49	0.28, 0.85	0.011	0.04	0.01, 0.25	<0.001	0.45	0.20, 1.03	0.059
Chronic kidney disease									
no	0.45	0.27, 0.75	0.002	—	—	—	—	—	—
TIMI after PCI									
0	—	—	—	—	—	—	—	—	—
1	—	—	—	1.42	0.11, 18.1	0.8	—	—	—
2	—	—	—	—	—	—	—	—	—
3	—	—	—	0.20	0.05, 0.81	0.024	—	—	—
Clinical status									
CCS	—	—	—	—	—	—	—	—	—
NSTEMI	0.93	0.53, 1.61	0.8	—	—	—	—	—	—
STEMI	1.73	0.99, 3.02	0.052	—	—	—	—	—	—
UA	0.37	0.15, 0.93	0.034	—	—	—	—	—	—
Left ventricular ejection fraction									
≤40	—	—	—	—	—	—	—	—	—
[40, 50]	0.53	0.32, 0.87	0.011	—	—	—	0.46	0.22, 0.98	0.044
[50, 60]	0.51	0.29, 0.90	0.020	—	—	—	0.24	0.09, 0.65	0.005
>60	0.43	0.23, 0.78	0.006	—	—	—	0.23	0.08, 0.69	0.008
LDL cholesterol									
≤100	—	—	—	—	—	—	—	—	—
[100, 129]	0.61	0.35, 1.06	0.080	—	—	—	—	—	—
[129, 159]	0.77	0.46, 1.29	0.3	—	—	—	—	—	—
[159, 465]	0.96	0.51, 1.81	>0.9	—	—	—	—	—	—

HR = hazard ratio; CCS—chronic coronary syndrome; CI = confidence interval; STEMI—ST-elevation myocardial infarction; NSTEMI—non-ST-elevation myocardial infarction.

**Table 7:** Outcomes in ACS patients—literature data.

Study	No of Patients	Comorbidities	Treatment	Outcomes
Grinberg et al. [18] High/very high/extremely high risk	5359 STEMI 39.1/32.8/23.9%	Hypertension: 87.7/92.4/97.3% DM: 57.1/69.4/80.7% Dyslipidemia: 74.1/77.5/82.3% Prior MI: 38.1/51.9/65.5% Prior PCI: 35.9/41.6/52.3%	PCI: 59.3/50.4/40.8% Based on time: 2002–2008 vs. 2010–2018: 52.6 vs. 66.5%/41.7 vs. 59.1%/34.7 vs. 47.2%	1-year mortality: 12.8/18.9/28.8%
Toyoda et al. [23]	3283 STEMI–68.9%	-	-	3-year mortality: STEMI: 14.9–16.9% NSTEMI: 5.6–29.6%
Piątek et al. [24] STEMI/NSTEMI/UA	STEMI: 2134 NSTEMI: 1162 UA: 2729	Hypertension: 62.7/70.8/76.6% DM: 18/21.3/24.7% Prior MI: 11/18.2/23.4% Prior PCI: 9.8/13.1/22.6%	All patients underwent PCI	3-year outcomes: Death: 15.4/15.5/10.5% MI: 3.5/2.2/1.6% PCI: 29.5/31.4/29.1% CABG: 3.2/2.5/2.0%
Reichlin et al. [27]	Acute MI: 242	Hypertension: 75% DM: 24% Dyslipidemia: 51% Prior MI: 31% Prior PCI: 28%	PCI/CABG: 68%	30-month mortality: 16.4–23.9% depending on MI size
Buller et al. [28] MINOCA/STEMI	MINOCA: 112 STEMI: 166	Hypertension: 53/42% DM: 13/17% Dyslipidemia: 25/37% Prior MI: 0/5.4% Prior PCI: 0/1.8%	All patients underwent PCI	9-year outcomes: All-cause death: 17.9/24.1% Cardiac death: 9.8/16.9% MI: 14.3/21.1%

STEMI—ST-elevation myocardial infarction; NSTEMI—non-ST-elevation myocardial infarction; MI—myocardial infarction; CABG—coronary artery bypass grafting; PCI—percutaneous coronary intervention; DM—diabetes mellitus; MINOCA—myocardial infarction with nonobstructive coronary arteries.

## Data Availability

Data are available from the corresponding author on request.

## Supplementary Materials

The following supporting information can be downloaded at https://www.mdpi.com/article/10.3390/biomedicines12122672/s1. Table S1: Univariable Cox regression for death in the CCS population. Table S2: Univariable Cox regression for death in ACS patients.
