# Supplementary material for: Comparative Ten-Year Outcomes in Chronic and Acute Coronary Syndrome Patients Undergoing Invasive Diagnostics—Insights from the KORONEF Registry
Source: Biomedicines. 2024 Nov 23;12(12):2672. doi: 10.3390/biomedicines12122672 (PMC11726982; doi:10.3390/biomedicines12122672)
Supplement: Supplementary file 1 [file biomedicines-12-02672-s001.zip › biomedicines-3315460-supplementary.pdf]

Supplementary Table S1. Cox regression for CCS subgroup.

	Univariable Cox regression			Multivariable Cox regression		
**Characteristic**	**HR[1]**	**95% CI**	**p-value**	**HR[1]**	**95% CI**	**p-value**
**Sex**						
F	—	—				
M	1.25	0.80, 1.94	0.3			
**Age - groups**						
(30,55]	—	—		—	—	
(55,60]	3.27	0.93, 11.5	0.064	5.29	0.63, 44.2	0.12
(60,65]	2.43	0.66, 8.97	0.2	3.04	0.33, 27.7	0.3
(65,75]	6.35	1.94, 20.8	0.002	3.97	0.48, 32.6	0.2
(75,90]	17.3	5.30, 56.4	<0.001	13.3	1.68, 106	0.014
**BMI**						
<25	—	—				
[25,29.9]	1.08	0.58, 2.02	0.8			
[30,34.9]	0.73	0.35, 1.52	0.4			
≥35	0.49	0.11, 2.18	0.4			
**Diabetes**						
Yes	—	—		—	—	
No	0.49	0.32, 0.74	<0.001	0.41	0.19, 0.87	0.021
**Hypertension**						
Yes	—	—				
No	0.78	0.44, 1.38	0.4			
**Smoking**						
Yes	—	—				
No	1.62	0.93, 2.83	0.087			
**Obesity**						
Yes	—	—				
No	1.13	0.72, 1.76	0.6			
**Dyslipidemia**						
Yes	—	—				
No	1.47	0.97, 2.25	0.071			
**Prior MI**						
Yes	—	—		—	—	
No	0.44	0.29, 0.66	<0.001	0.46	0.22, 0.98	0.045
**Prior stroke**						
Yes	—	—				
No	0.40	0.22, 0.73	0.003			
**PAD**						
Yes	—	—				
No	0.67	0.32, 1.38	0.3			
**Dialysis**						
Yes	—	—				
No	9,121,182	0.00, Inf	>0.9			
**CKD**						
Yes	—	—				
No	0.33	0.20, 0.55	<0.001			
**Prior CABG**						
Yes	—	—				

Supplementary Table S1. Cox regression for CCS subgroup.

	Univariable Cox regression			Multivariable Cox regression		
**Characteristic**	**HR[1]**	**95% CI**	**p-value**	**HR[1]**	**95% CI**	**p-value**
No	0.58	0.28, 1.21	0.15			
**Prior PCI**						
Yes	—	—				
No	0.70	0.45, 1.11	0.13			
**AF**						
Yes	—	—		—	—	
No	0.36	0.19, 0.70	0.002	0.04	0.01, 0.25	<0.001
**CAD advancement**						
3-VD	—	—				
LM	1.02	0.40, 2.63	>0.9			
**BMS No**						
0	—	—				
1	1.08	0.62, 1.88	0.8			
2	3.22	1.17, 8.81	0.023			
3						
**DES No**						
0	—	—				
1	0.84	0.41, 1.74	0.6			
2	0.73	0.10, 5.27	0.8			
3	0.00	0.00, Inf	>0.9			
**POBA**						
0	—	—				
1	0.94	0.47, 1.87	0.9			
2						
**TIMI after PCI**						
0	—	—		—	—	
1	0.48	0.05, 4.71	0.5	1.42	0.11, 18.1	0.8
2						
3	0.12	0.04, 0.42	<0.001	0.20	0.05, 0.81	0.024
**RAS**						
<50%	—	—				
≥50%	1.58	0.79, 3.15	0.2			
**Echo EF**						
≤40	—	—				
(40,50]	0.65	0.35, 1.21	0.2			
(50,60]	0.67	0.35, 1.29	0.2			
>60	0.32	0.16, 0.64	0.001			
**hsCRP**						
≤0.1	—	—				
(0.1,0.2]	0.82	0.44, 1.55	0.5			
(0.2,0.5]	1.08	0.60, 1.92	0.8			
(0.5,82]	1.75	0.95, 3.20	0.071			
**LDL chol**						
≤100	—	—				
(100,129]	0.41	0.22, 0.79	0.007			

Supplementary Table S1. Cox regression for CCS subgroup.

	Univariable Cox regression			Multivariable Cox regression		
**Characteristic**	**HR[1]**	**95% CI**	**p-value**	**HR[1]**	**95% CI**	**p-value**
(129,159]	0.70	0.37, 1.30	0.3			
(159,465]	1.05	0.52, 2.12	0.9			
**Glucose**						
≤80	—	—				
(80,100]	0.69	0.25, 1.96	0.5			
(100,140]	1.20	0.43, 3.37	0.7			
(140,200]	1.11	0.34, 3.62	0.9			
>200	1.48	0.37, 5.92	0.6			
**Creatinine clearens**						
≤60	—	—				
>60	0.32	0.21, 0.51	<0.001			

[1]HR = Hazard Ratio, CI = Confidence Interval

Supplementary Table S2. Cox regression for ACS subgroup.

	Univariable Cox regression			Multivariable Cox regression		
**Characteristic**	**HR[1]**	**95% CI[1]**	**p-value**	**HR[1]**	**95% CI[1]**	**p-value**
**Sex**						
F	—	—				
M	0.69	0.40, 1.21	0.2			
**Age - group**						
(30,55]	—	—		—	—	
(55,60]	1.43	0.48, 4.27	0.5	0.94	0.30, 2.97	>0.9
(60,65]	0.89	0.24, 3.30	0.9	0.54	0.13, 2.34	0.4
(65,75]	2.41	0.88, 6.63	0.089	1.60	0.54, 4.69	0.4
(75,90]	6.50	2.39, 17.7	<0.001	4.99	1.70, 14.7	0.003
**BMI**						
<25	—	—				
[25,29.9]	0.66	0.32, 1.39	0.3			
[30,34.9]	1.00	0.42, 2.39	>0.9			
≥35	1.01	0.29, 3.53	>0.9			
**Diabetes**						
Yes	—	—				
No	0.54	0.30, 0.98	0.044			
**Hypertension**						
Yes	—	—				
No	1.00	0.56, 1.78	>0.9			
**Smoking**						
Yes	—	—				
No	1.61	0.87, 2.99	0.13			
**Obesity**						
Yes	—	—				
No	0.86	0.46, 1.61	0.6			
**Dyslipidemia**						
Yes	—	—				
No	1.50	0.83, 2.69	0.2			
**Prior MI**						
Yes	—	—				
No	0.57	0.31, 1.05	0.072			
**Prior stroke**						
Yes	—	—		—	—	
No	0.33	0.14, 0.77	0.011	0.27	0.11, 0.68	0.005
**PAD**						
Yes	—	—				
No	0.34	0.12, 0.94	0.039			
**Dialysis**						
Yes	—	—				
No						
**CKD**						
Yes	—	—				
No	0.47	0.21, 1.05	0.065			
**Prior CABG**						
Yes	—	—				

Supplementary Table S2. Cox regression for ACS subgroup.

	Univariable Cox regression			Multivariable Cox regression		
**Characteristic**	**HR[1]**	**95% CI[1]**	**p-value**	**HR[1]**	**95% CI[1]**	**p-value**
No	0.77	0.11, 5.55	0.8			
**Prior PCI**						
Yes	—	—				
No	1.04	0.52, 2.08	>0.9			
**STEMI**						
Yes	—	—				
No	0.86	0.49, 1.50	0.6			
**NSTEMI**						
Yes	—	—				
No	0.78	0.44, 1.40	0.4			
**UA**						
Yes	—	—				
No	1.79	0.65, 4.98	0.3			
**Cardiac arrest**						
Yes	—	—		—	—	
No	0.29	0.09, 0.93	0.037	0.20	0.04, 0.95	0.044
**AF**						
Yes	—	—		—	—	
No	0.42	0.20, 0.90	0.026	0.45	0.20, 1.03	0.059
**Disease Advancement**						
3-VD	—	—				
LM	2.78	0.73, 10.6	0.13			
**BMS No**						
0	—	—				
1	1.01	0.55, 1.85	>0.9			
2	1.45	0.59, 3.56	0.4			
3	2.07	0.49, 8.82	0.3			
**DES No**						
0	—	—				
1	0.47	0.22, 1.00	0.051			
2	0.71	0.10, 5.14	0.7			
3						
**POBA**						
0	—	—				
1	0.89	0.48, 1.64	0.7			
2	2.80	0.38, 20.5	0.3			
**TIMI after PCI**						
0	—	—				
1	0.00	0.00, Inf	>0.9			
2	0.00	0.00, Inf	>0.9			
3	0.63	0.20, 2.05	0.4			
**RAS**						
<50%	—	—				
≥50%	1.16	0.46, 2.91	0.8			

Supplementary Table S2. Cox regression for ACS subgroup.

	Univariable Cox regression			Multivariable Cox regression		
**Characteristic**	**HR[1]**	**95% CI[1]**	**p-value**	**HR[1]**	**95% CI[1]**	**p-value**
**Echo EF**						
≤40	—	—		—	—	
(40,50]	0.40	0.20, 0.81	0.011	0.46	0.22, 0.98	0.044
(50,60]	0.20	0.08, 0.51	<0.001	0.24	0.09, 0.65	0.005
>60	0.18	0.06, 0.50	0.001	0.23	0.08, 0.69	0.008
**hsCRP**						
≤0.1	—	—				
(0.1,0.2]	2.02	0.54, 7.62	0.3			
(0.2,0.5]	1.56	0.45, 5.43	0.5			
(0.5,82]	2.01	0.60, 6.73	0.3			
**LDL chol**						
≤100	—	—				
(100,129]	0.65	0.29, 1.46	0.3			
(129,159]	0.61	0.28, 1.32	0.2			
(159,465]	0.66	0.29, 1.48	0.3			
**Glucose**						
≤80	—	—				
(80,100]	3,797,915	0.00, Inf	>0.9			
(100,140]	3,705,647	0.00, Inf	>0.9			
(140,200]	4,004,548	0.00, Inf	>0.9			
>200	12,740,871	0.00, Inf	>0.9			
**Creatinine clearance**						
≤60	—	—				
>60	0.31	0.17, 0.58	<0.001			

[1]HR = Hazard Ratio, CI = Confidence Interval
